# SepM mutation in *Streptococcus mutans* clinical isolates and related function analysis

**DOI:** 10.1186/s12903-024-04436-x

**Published:** 2024-06-25

**Authors:** Shanshan Liu, Yidan Shao, Zhenzhen Zhang, Wen Xu, Yudong Liu, Kai Zhang, Li Xu, Qingwei Zheng, Yu Sun

**Affiliations:** 1 Department of Stomatology, The First Affiliated Hospital of Bengbu Medical College, 287 Chuang Huai Road, Bengbu, 233004 China; 2 Anhui Key Laboratory of Infection and Immunity, Bengbu Medical College, 2600 Dong Hai Avenue, Bengbu, 233030 China; 3 Department of Stomatology, Bengbu Medical College, 2600 Dong Hai Avenue, Bengbu, 233030 China; 4 Department of Biochemistry and Molecular Biology, Bengbu Medical College, 2600 Dong Hai Avenue, Bengbu, 233030 China; 5 Department of Histology and Embryology, Bengbu Medical College, 2600 Dong Hai Avenue, Bengbu, 233030 China

**Keywords:** Gene polymorphism, Expression, SepM, Caries

## Abstract

**Background:**

*Streptococcus mutans* (*S. mutans*) is an important pathogenic bacterium that causes dental caries, while *Streptococcus gordonii* (*S. gordonii*) is a non-cariogenic bacterium that inhibits the growth of *S. mutans*. The SepM protein can promote the inhibitory ability of *S. mutans* against *S. gordonii* by cleaving CSP-21 and activating the ComDE two-component system. This study was designed to explore *sepM* mutation in *S. mutans* clinical isolates and related function in the regulation of interactions with *S. gordonii*.

**Methods:**

The *S. mutans* clinical strains that can inhibit the growth of *S. gordonii* constitute the inhibitory group. 286 C-serotype *S. mutans* strains were categorized into *S. gordonii* inhibitory (*n* = 114) and non-inhibitory bacteria (*n* = 172). We detected sanger sequencing of *sepM* gene, the expression levels of related genes and proteins in clinical isolates, obtained prokaryotic expression and purification of mutated proteins, and analyzed the effect of the target mutations on the binding between SepM and CSP-21.

**Results:**

We found that C482T, G533A, and G661A missense mutations were presented at significantly higher frequency in the inhibitory group relative to the non-inhibitory group. There was no significant difference in the expression of the *sepM* gene between selected clinical isolates harboring the G533A mutation and the control group. The expression levels of SepM, phosphorylated ComD, and ComE in the mutation group were significantly higher than those in the control group. SepM_control and SepM_D221N (G661A at the gene level) were found to contain two residues close to the active center while SepM_G178D (G533A at the gene level) contained three residues close to the active center. At 25 °C and a pH of 5.5, SepM_D221N (G661A) exhibited higher affinity for CSP-21 (KD = 8.25 µM) than did the SepM control (KD = 33.1 µM), and at 25 °C and a pH of 7.5, SepM_G178D (G533A) exhibited higher affinity (KD = 3.02 µM) than the SepM control (KD = 15.9 µM). It means that it is pH dependent.

**Conclusions:**

Our data suggest that increased cleavage of CSP-21 by the the mutant SepM may be a reason for the higher inhibitory effect of *S. mutans* on *S. gordonii* .

**Supplementary Information:**

The online version contains supplementary material available at 10.1186/s12903-024-04436-x.

## Background

In addition to being a highly prevalent infectious bacterial disease, dental caries can have a serious adverse impact on patient quality of life and can lead to potentially severe complications including pain or tooth loss [1–6]. *Streptococcus mutans* (*S. mutans*) is a major cariogenic bacterial species that facilitates local acidification at tooth surfaces by metabolizing carbohydrates present in the oral microenvironment [7]. When the local pH falls below 5.5, this can lead to the demineralization of the tooth surface and the consequent development of dental caries [8].

*Streptococcus gordonii* (*S. gordonii*) is a Gram-positive bacterium that is thought to be a major initial colonizing species involved in the formation of dental plaque biofilms [9, 10]. *S. gordonii* metabolic activity can lead to the production of H_2_O_2_ [11], which inhibits *S. mutans* growth, as well as the production of ammonia which can counteract local tooth surface acidification to protect against the onset or progression of cariogenesis [12]. Previously reported research studies have highlighted a positive correlation between *S. mutans* detection rates in dental plaque and dental caries incidence [13, 14], whereas *S. gordonii* is negatively correlated with dental caries [15, 16].

Bacteriocins are a family of ribosomally synthesized peptide antibiotics that are produced by bacteria that resist adverse environments [17]. The bacteriocin produced by *S. mutans* is called mutacin [17, 18]. It has been reported that *S. mutans* produces several types of mutacins including mutacins I-IV, K8 and Smb. Mutacin IV positive *S. mutans* strains has been shown to readily suppress *S. gordonii* growth [19, 20]. *S. mutans* secretes mutacin IV, which is a bacteriocin encoded by *nlmA* and *nlmB* and regulated by the ComDE two-component system, enabling it to effectively inhibit *S. gordonii* microbial activity [21, 22]. In in vivo plaque biofilms, Tanzer et al. found that *S. mutans* was capable of outcompeting *S. gordonii* and was highly cariogenic [23]. CSP-21 is a 21-residue competence-stimulating peptide that can interact with the membrane-bound histidine kinase receptor ComD, leading to its autophosphorylation and the transfer of the phosphate group to its cognate cytoplasmic response regulator ComE, which is then capable of binding to the promoter regions upstream of genes encoding mutacin IV (*nlmA* and *nlmB*), promoting its upregulation [24]. SepM cleaves CSP-21 into CSP-18, which is more effective in activating the ComDE system than CSP-21 [25]. It is known that mutacin IV inhibits *S. gordonii* growth, and many studies on mutacin IV have used *S. gordonii* as an indicator bacterium [17, 21]. Therefore, SepM is a key factor in regulating the inhibitory effect of *S. mutans* on *S. gordonii*.

In *S. mutans*, the SepM protein is 346 amino acids in length and includes a minimum of one transmembrane domain (amino acids 10–26), a eukaryotic-type PDZ domain (amino acids 131–195), and a C-terminal Lon-like protease (S16) domain (amino acids 233–314) [26]. PDZ domains are most commonly present in multicellular organisms wherein they function as common modules that facilitate interactions between proteins through the recognition of short peptide sequences most often present in the C-terminal regions of proteins associated with the plasma membrane. These domains contribute to protein complex formation and spatial confinement, thereby exhibiting ligand specificity and regulating cellular signaling processes [27, 28]. The C-terminal domains of serine proteases also contain the residues that form the active site for substrate binding and the regulation of catalytic activity. Given that genetic mutations can impact virulence and thereby enhance or lower disease-related risk, the present study was developed to analyze *sepM* gene mutations of the PDZ and C-terminal domains across 286 serotype C *S. mutans* clinical isolates and explore the role that these mutations play in governing interactions between *S. mutans* and *S. gordonii*.

## Methods

### Biological phenotype

This study received ethical approval from the ethics committee of the First Affiliated Hospital of Bengbu Medical College (Approval No [2017] KY011) for the *S. mutans* clinical isolates collection. Informed consent was obtained from all subjects and/or their legal guardian(s). These 286 isolates were collected from samples of dental plaque obtained from 3-6-year-old children with or without dental caries that are preserved in our laboratory. The isolation and serotype c identification methods for these *S. mutans* clinical isolates, as well as the activity of these isolates against *S. gordonii*, were detailed in our prior study [29, 30]. All sequences of the 286 *S. mutans* clinical isolates were submitted to the National Center for Biotechnology Information (NCBI) under the BioProject accession number PRJNA1016128. Briefly, plaque samples were centrifuged for 30 s, plated 50 µL of saline on Trypticase Yeast-Extract Cysteine Sucrose Bacitracin (TYCSB) agar, and then cultured for 48 h at 37 °C under 5% CO_2_. Clinical *S. mutans* were confirmed according to their reaction to mannitol, sorbitol, raffinose, melibiose, aesculin, arginine hydrolase and arginine hydrolase control [31]. Then all isolates were cultured overnight in brain heart infusion (BHI) broth and then DNA was extracted from these isolates using a Bacterial Genome DNA Extraction Kit (Tiangen Biotech, Beijing, China) according to the manufacturer’s instructions. The confirmation of the C serotype was carried out by polymerase chain reaction (PCR) using serotype-specific primers. The PCR conditions for C serotype were as follows: denaturation at 96 °C for 2 min; 25 cycles consisting of 15 s of denaturation at 96 °C; 30 s of annealing at 61 °C; and 1 min of extension at 72 °C. The sequences of PCR primers for the identification of serotypes are listed in Table 1 [32]. The amplification products were then observed through electrophoresis in agarose gels. *S. mutans* UA159 was used as a positive reference. Bacteriocin assay was used to evaluate the activity of *S. mutans* against *S. gordonii*. Each isolate was incubated overnight in BHI broth, and then 10 µl of each clinical isolate with an OD600 of 0.3 were added to BHI agar. Equal amounts of *S. gordonii* (ATCC 10,558) were inoculated adjacent to the *S. mutans* sample after 12 h of incubation. The agar plate medium was then incubated for another 12 h. *S gordonii* clearing zones represent *S. mutans*’s activity against *S. gordonii*. The bacteriocin assay was conducted in three biological replicates.

**Table 1:** Primers used for the present study

ID	Sequence (5’→3’)	Size of the PCR product (bp)
Serotype *c*-F ^[32]^	CGGAGTGCTTTTTACAAGTGCTGG	727
Serotype *c*-R ^[32]^	AACCACGGCCAGCAAACCCTTTAT	
Serotype *e*-F ^[32]^	CCTGCTTTTCAAGTACCTTTCGCC	517
Serotype *e*-R ^[32]^	CTGCTTGCCAAGCCCTACTAGAAA	
Serotype *f*-F ^[32]^	CCCACAATTGGCTTCAAGAGGAGA	316
Serotype *f*-R ^[32]^	TGCGAAACCATAAGCATAGCGAGG	
Serotype *k*-F ^[32]^	ATTCCCGCCGTTGGACCATTCC	294
Serotype *k*-R ^[32]^	CCAATGTGATTCATCCCATACC	
*sepM*-F_mutation	GTGAAAACAAACAAAAAATTTAAAT	1041
*sepM*-R_mutation	TTAGTGTTTTCTTAGGTAATCAATA	
*sepM*-F_expression	GCAGCAAGGTCAGTGTTCAA	191
*sepM*-R_expression	GGTAAACATGAGACCGGCAC	
*comD*-F	GCCTGAGATGGAGTTGCTTG	217
*comD*-R	GCGATTGGAGCCTTTAGTGG	
*comE*-F	CCTGAAAAGGGCAATCACCA	232
*comE*-R	CTGATTCAATGCGGTGGGAG	
*nlmA*-F	GGACAGCCAAACACTTTCAAC	156
*nlmA*-R	ATGAGTCCCCAAGTGCCTAC	
*nlmB*-F	TTTTGGTGGAGATAAACAAGCTG	150
*nlmB*-R	AAAACTACAGATCCAACCGCA	
16s rRNA-F	CTGACTTGAGTGCAGAAGGGGA	109
16s rRNA-R	CGTCAGTGACAGACCAGAGAGC	

**Table 2:** The relationship between *sepM* mutations and the virulence of *S. mutans* against *S. gordonii*

Gene mutation	Reference codon	Mutation codon	Amino mutation	Mutation type	Distribution of mutation (*n*, %)		
					Inhibitory group (*n* = 114)	Non-Inhibitory group (*n* = 172)	*P* value
T392G	ATG	AGG	M131R	Missense	0 (0.0)	1 (0.6)	0.999
G412A	GTT	ATT	V138I	Missense	1 (0.9)	0 (0.0)	0.399
G419A	AGG	AAG	R140K	Missense	110 (96.5)	171 (99.4)	0.084
C482T	ACC	ATC	T161I	Missense	5 (4.4)	1 (0.6)	0.039
C483T	ACC	ACT	T161T	Synonymous	0 (0.0)	2 (1.2)	0.519
T493A	TCA	ACA	S165T	Missense	1 (0.9)	0 (0.0)	0.399
G516C	GTG	GTC	L172V	Missense	0 (0.0)	2 (1.2)	0.519
G533A	GGC	GAC	G178D	Missense	29 (25.4)	12 (7.0)	< 0.001
G603A	TTG	TTA	L201L	Synonymous	1 (0.9)	0 (0.0)	0.399
A614C	AAA	ACA	K205T	Missense	3 (2.6)	0 (0.0)	0.062
G661A	GAT	AAT	D221N	Missense	6 (5.3)	0 (0.0)	0.004
G694A	GGA	AGA	G232R	Missense	0 (0.0)	1 (0.6)	0.999
G826A	GGT	AGT	G276S	Missense	0 (0.0)	2 (1.2)	0.519
C831T	GCC	GCT	A277A	Synonymous	1 (0.9)	0 (0.0)	0.399
C845T	GCA	GTA	A282V	Missense	0 (0.0)	2 (1.2)	0.519
C908T	ACT	ATT	T303I	Missense	22 (19.3)	42 (24.4)	0.309

### *sepM* polymorphism analyses

*S. mutans* DNA was extracted from the clinical isolates at stationary phase as in our prior study [33], after which it was used as a template for PCR reactions. PCR primers (*sepM*-F_mutation and *sepM*-R_mutation) were designed with Primer Premier 5.0 to amplify the DNA fragment corresponding to the *sepM* PDZ domain and C-terminal domain. Thermocycler settings were as follows: 5 min at 94 °C; 35 cycles of 94 °C for 30 s, 55 °C for 40 s and 72 °C for 30 s; and 72 °C for 5 min. The resultant DNA was analyzed by 1% agarose gel electrophoresis, and was subsequently sequenced by HuaXiao gene technology (Bengbu, China) with an ABI3730 instrument.

### RNA extraction and qRT-PCR

Total RNA was extracted using 25 mg/mL lysozyme (TIANGEN Biotechnology, China) for 30 min and the RNeasy Mini Kit (Qiagen), and was then reverse transcribed into cDNA using the PrimeScript™ RT reagent Kit (Takara). The qRT- PCR of analyses of *sepM*, *comD*, *comE*, *nlmA*, and *nlmB* gene expression was performed using a 20 µL volume containing 10 µL of 2× SYBR Premix Ex TaqII, 1 µL each of the forward and reverse primers, 1 µL of cDNA, and 7 µL of RNase-free H_2_O. The amplification program settings were as follows: 95 °C for 30 s; 40 cycles of 95 °C for 5 s and 55 °C for 25 s. At the end of the reaction, a melting curve was generated. The sequences of PCR primers are listed in Table 1.

### Protein extraction and Western blotting

Bacteria at the stationary phase were collected by centrifugation (15 min, 8,000 rpm) and washed using PBS. Lysozyme (TIANGEN Biotechnology, China) was diluted with lysozyme buffer (SL20734, COOLABER SCIENCE & TECHNOLOGY, China). This buffer was composed of 20 mM Tris (pH 8.0), 2 mM sodium Na2-EDTA and 1.2% Triton X-100. To lyse these cells, 100 µL of 25 mg/mL lysozyme was added per tube followed by incubation for 30 min at 37 °C, followed by the addition of 300 µL of RIPA buffer (P0013B, Beyotime Biotechnology, China), and incubation for 10 min on ice. The main composition of RIPA buffer was 50 mM Tris (pH 7.4), 150 mM NaCl, 1% Triton X-100, 1% sodium deoxycholate, 0.1% SDS, sodium orthovanadate, sodium fluoride, EDTA and leupeptin. Samples were then centrifuged, and the supernatant was collected. A BCA Protein Assay Kit (Beyotime, China) was then used to measure protein concentrations in each sample and to dilute samples to 1 mg/mL [34]. Proteins were then combined with loading buffer, heated to 100 ℃ and boiled for 5 min, and subsequently stored at -20 ℃. Following SDS-PAGE separation these proteins were transferred to PVDF membranes (Sigma, MO, USA) that were blocked for 1 h using 5% skim milk in TBS containing 0.1% Tween-20 (TBST) at room temperature. After three washes with TBST, blots were probed overnight with antibodies specific for SepM, ComD, or ComE (diluted 1:500) at 4 ℃, rinsed with TBST, and incubated for 1 h with HRP-conjugated goat anti-Mouse IgG (diluted 1:2,000; S0002, Affinity Biosciences, Jiangsu, China) for SepM or HRP-conjugated Affinipure Goat Anti-Rabbit IgG (H + L) (diluted 1:2,000; SA00001-2, Proteintech, Wuhan, China) for ComD and ComE at room temperature. An ECL Western Blotting Substrate Kit (Cat: #KF005, Affinity Biosciences, Jiangsu, China) was used to detect protein bands. The antibodies specific for SepM, ComD, or ComE were made by HUABIO (Hangzhou, China). The brief steps are as follows: (1) Recombinant SepM, ComD, and ComE was expressed and purified; (2) Antibodies were prepared by immunizing New Zealand white rabbits three times at two-week intervals with 100 µg eluted SepM, ComD or ComE protein that was homogenized in complete Freund’s adjuvant; (3) A boost injection was given one week later; (4) Rabbit serum was collected seven days after the final immunization and stored at − 80 °C. The extraction steps of phosphorylated proteins were similar to those of conventional protein extraction, except that after adding RIPA buffer, 10 µL of Protease Inhibitor Cocktail (P1025, Beyotime Biotechnology, China) needed to be added for co incubation. For phosphorylated proteins expression analysis, a 10% phosphorylated gel was prepared using Beyotime SDS-PAGE Gel Preparation Reagent Kit (beyotime, Shanghai, China, CatNo.P0012A) and Phosbind Acrylamide (APExBIO Houston, USA, CatNo. F4002). 10 µL of the target protein sample and 2.5 µL of Prestained Protein Marker (APExBIO Houston, USA, Cat No. F4005) are loaded onto the gels. ComD antibody and Proteintech Affinipure Goat Anti-Rabbit IgG (H + L) were diluted in a 5% BSA (Solarbio, Beijing, China, Cat No. A8020) at a ratio of 1:250. The other steps were the same as routine Western blotting.

### Molecular docking and molecular dynamics simulations

SepM protein crystal structures were not present in the RCSB Protein Data Bank (http://www.pdb.org/). The SepM protein structure was therefore predicted using Iterative Threading ASSEmbly Refinement (I-TASSER) (http://zhanglab.ccmb.med.umich.edu/I-TASSER/output/S689586/) [35, 36] followed by modification with the Schrodinger 2019.01 Protein Preparation Wizard model, including water molecule removal, hydrogen addition, amino acid optimization, and patching. Protein-substrate complex stability and dynamics were assessed through a slightly modified version of a previously reported molecular dynamics simulation approach [37]. Briefly, the simulation box was solvated with TIP3P water molecules together with appropriate counter ions to neutralize the system. System energy was minimized using optimized parameters for liquid simulation (OPLS3e) forcefield. In total, 100 ns of equilibration simulations were run in the NpT ensemble using the following parameters: temperature, 300 K; pressure,1.0 bar; integration time step, 1.2 fs. All bonds involving hydrogen atoms were constrained with the SHAKE method. Docking analyses on SepM and CSP-21 were performed with HDOCK (http://hdock.phys.hust.edu.cn/) [38], and the top-ranked docking model was visualized with PyMOL 2.1.

### SepMs expression and purification

Recombinant SepM_control, SepM_G178D, and SepM_D221N were purchased from Yangene Biological Technology (Wuhan, China). The brief steps are as follows: (1) Synthesized SepMs (SepM_control, SepM_G178D, and SepM_D221N) sequences were ligated into the pET-sumo vector, respectively. (2) The plasmid was validated by sequencing and subsequently used to transform *E. coli* BL21 (DE3) (Novagen, USA). (3) Following short-term culture and isopropyl-β-D-thiogalactopyranoside (IPTG) induction (0.8 mM) at 16 ℃ for 15 h, cells were collected by centrifugation (10 min, 4,000 rpm). (4) Pellets were resuspended in lysis buffer and disrupted by sonication on ice (30 min, 300 W work 5s-off 3s). (5) Lysates were then centrifuged at 18,000 rpm under 4 °C for 15 min. (6) The supernatant was then collected and incubated with Ni resin at 4 °C for 2 h. (7) The column was washed with a gradient of concentrations of imidazole solution. (8) 10 mL of elution buffer was added to the column to elute the bound proteins. (9) Recombinant proteins were then analyzed by 15% SDS-PAGE.

### Microscale thermophoresis (MST) assay

SepM_control, SepM_G178D, and SepM_D221N binding to CSP-21 were monitored through an MST approach [39]. CSP-21 was synthesized by GenScript (Nanjing, China). These MST assays were conducted using a Monolith NT.115 instrument (NanoTemper Technologies GmbH, Munich, Germany). SepM_control, SepM_G178D, and SepM_D221N (final concentration, 50 nM) possessed a His-tag were mixed with His-Tag Labeling Kit-RED-tris-NTA (MO-L018, NanoTemper Technologies) at 25 °C for 30 min in the dark, and then individually mixed with CSP-21 (initial concentration, 100 µM) in a 16-point serial dilution series. The interactions between the proteins and CSP-21 were measured in Monolith NT.115 Standard Treated Capillaries (NanoTemper Technologies). Samples were prepared in PBS at a pH of 5.5 or 7.5. Measurements were made at 37 °C and 25 °C using 50% light-emitting diode (LED) power and medium MST power. Experiments were repeated in triplicate for all measurements. The KD values were analyzed using MO Affinity Analysis 2.3 software.

### Statistical analysis

SPSS 22.0 (IBM, NY, USA) was used to examine the relationship between the inhibition of *S. mutans* on *S. gordonii* and *sepM* genetic polymorphisms, and the expression levels of genes between groups of mutation and mutation_free. Qualitative data were analyzed using the Pearson chi-square test for a theoretical frequency ≥ 5. For theoretical frequencies between 1 and 5, data were analyzed via continuity correction. All other results were analyzed with Fisher’s exact test. The Shapiro-Wilk test was used to assess whether the quantitative data were parametric. For parametric testing, a t test was used, for nonparametric testing, the Mann-Whitney U test was used. *P* < 0.05 was the threshold for statistical significance.

## Results

We aimed to explore whether there is a *sepM* gene mutation in *S. mutans* clinical strains that regulates the ability of *S. mutans* to inhibit the growth of *S. gordonii*. The *S. mutans* clinical strains that can inhibit the growth of *S. gordonii* were defined as an inhibitory group, otherwise they are considered as the non-inhibitory group. When the *sepM* gene sequences from these 286 clinical isolates were compared to the reference *S. mutans* UA159 sequence, 16 single nucleotide polymorphisms were identified, including 3 silent mutations and 13 missense mutations. None of the sequenced *sepM* genes exhibited base insertions or deletions. Significant differences in mutation frequencies were observed at loci 482, 533, and 661 when comparing the inhibitory and non-inhibitory groups (*P* = 0.039, < 0.001, and 0.004, respectively), with all three of these sites being more frequently mutated in the antagonistic group (Table 2). Fig. S1 showed the results of *S. mutans* inhibiting the growth of *S. gordonii* and the serotype validation of *S. mutans*.

**Fig. 1:**
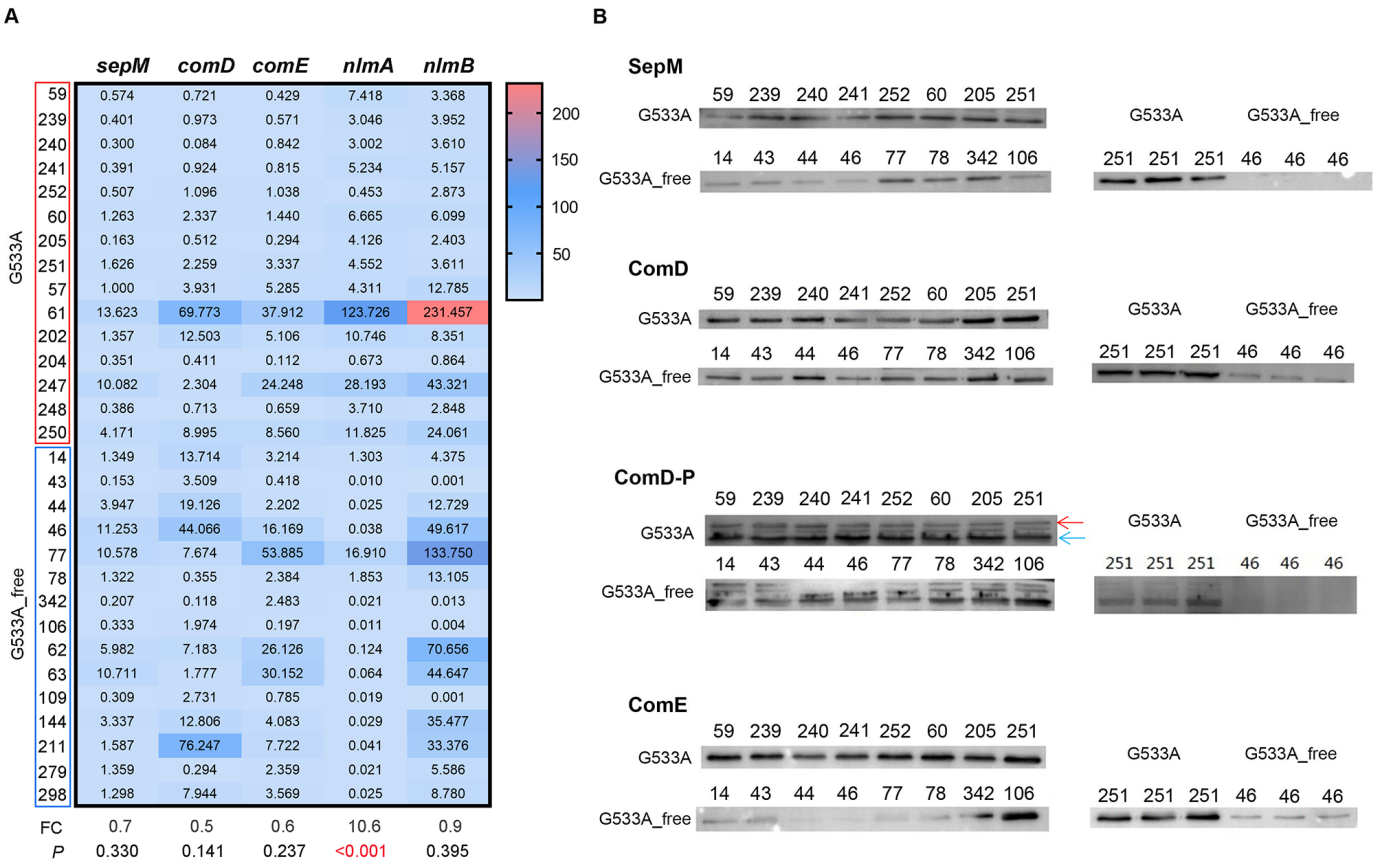
Gene and protein expression levels in *S. mutans* clinical isolates. **(A)** Relative *sepM*, *comD*, and *comE*, *nlmA* and *nlmB* gene expression levels were compared between groups that did and did not harbor the G533A mutation. **(B)** The expression levels of SepM, ComD, ComE, and phosphorylated ComD compared across 16 isolates. To better display the differences between groups, we displayed samples from the same group on a piece of glue, we also presented a pair of inter group samples on a piece of glue to reduce the impact of exposure factors on the comparison of inter group samples. ComD-P represents phosphorylated ComD. The red arrow represents phosphorylated ComD, and the blue arrow represents unphosphorylated ComD

To examine the role that the *sepM* G533A mutation in the ability of *S. mutans* to inhibit *S. gordonii*, the levels of *sepM* and downstream genes were analyzed in 30 selected clinical isolates including 15 harboring the G533A mutation (G533A group) and 15 control isolates lacking this mutation (G533A_free group). Isolates in the G533A group were selected from the inhibitory group which can inhibit the growth of *S. gordonii*, while the bacteria in the G533A_free group were selected from non-inhibitory group. Overall, 98% of the analyzed isolates (281/286) shared the G419A mutation, so the G419A point mutation was present in all 30 selected isolates, but with no interference of other *sepM* silent or missense mutation sites in these isolates. We found that *nlmA* was expressed at significantly (*P* < 0.001) higher levels in the G533A group relative to the control group, while no differences between these groups were observed with respect to *sepM*, *comD*, *comE*, or *nlmB* expression levels (*P* = 0.330, 0.141, 0.237, and 0.395, respectively) (Fig. 1A). We subsequently assessed the expression levels of SepM, ComD, ComE, phosphorylated ComD, and phosphorylated ComE in 16 clinical isolates with G533A mutation (G533A group, *n* = 8) and lacking G533A mutation (control group, *n* = 8). These isolates were selected randomly from the isolates in mRNA analysis. We found SepM and ComE protein levels were higher in the G533A group relative to the control group; although ComD protein levels did not differ substantially between these groups but phosphorylated ComD protein levels were higher in the G533A group relative to the control group (Fig. 1B). Full-length gels of Fig. 1B, Fig S1 A and Fig S2 were included in Supplementary material. Phosphorylated ComE failed detection after multiple experiments. Fig S3 showed the reaction of clinical strains with mannitol, sorbitol, raffinose, melibiose, aesculin, arginine hydrolase and arginine hydrolase control.

**Fig. 2:**
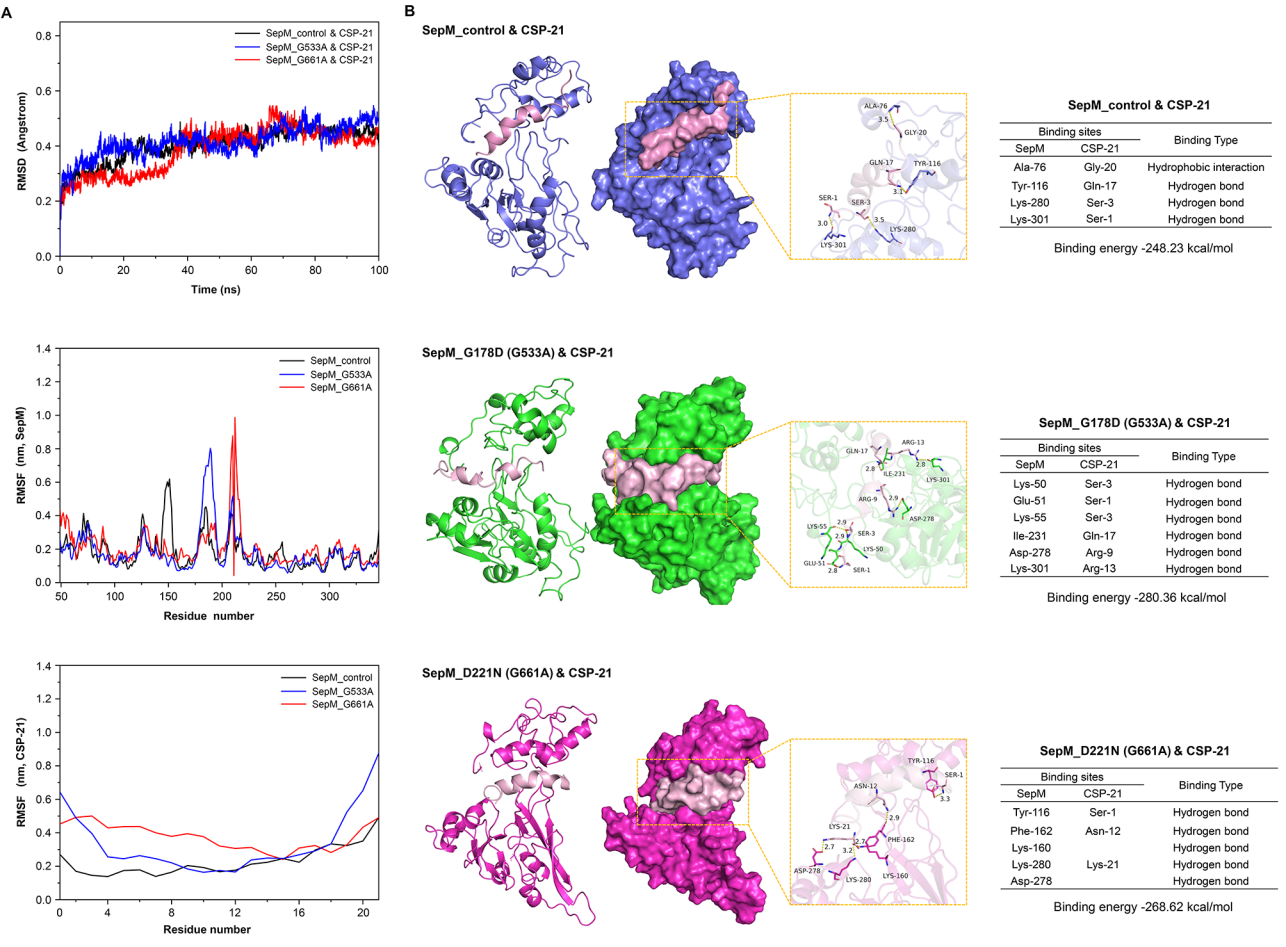
The binding mode for interactions between SepMs and CSP-21. **(A)** A root-mean-square displacement (RMSD) plot, a root-mean-square fluctuation (RMSF) plot for SepM and a RMSF plot for CSP-21. RMSF diagrams demonstrated that the structure of the SepM_G533A-CSP-21 complex exhibited more fluctuations at 175–200 residues relative to other binding sites, the structure of the SepM_G661A-CSP-21 complex had more fluctuations at 220–225 residues relative to other binding sites. This suggests mutations in SepM exhibit effective fluctuations conducive to its binding with CSP-21; **(B)** The binding mode for interactions between SepM_control and CSP-21

Mutation of G533A at the gene level corresponds to G178D, the latter is a change at amino acid level. Mutation of G661A at the gene level corresponds to D221N at the amino acid level. Potential binding sites and stability were also compared among SepMs (SepM_control, SepM_G178D (G533A), SepM_D221N (G661A)) and CSP-21 through molecular docking and molecular dynamics simulation approaches. A root-mean-square displacement (RMSD) cutoff of 2Å is frequently used as a criterion when predicting correct bond structures. The RMSD value of < 0.6 in this study indicated that the SepM-CSP-21 complex was stable (Fig. 2A). Root-mean-square fluctuation (RMSF) values further demonstrated that the structure of this SepM_G533A-CSP-21 complex exhibited more fluctuations for 175–200 residues relative to other binding sites, the structure of the SepM_G661A-CSP-21 complex had more fluctuations for 220–225 residues relative to other binding sites. This indicates that mutations can affect the binding mode through which SepM and CSP-21 interact. All binding energy values were below − 200, indicating a stable binding interaction between all three analyzed SepM isoforms and CSP-21. Identified binding sites between SepM_control and CSP-21 included ALA-76, TYR-116, LYS-280, and LYS-301, while binding sites between SepM_D221N and CSP-21 included TYR-116, PHE-162, LYS-160, ASP-278, and LYS-280, and binding sites between SepM_G178D and CSP-21 included LYS-50, GLU-51, LYS-55, ILE-231, ASP-278, and LYS-301 (Fig. 2B). Of these complexes, SepM_G178D exhibited three residues (ILE-231, ASP-278, and LYS-301) that were very close to the active center (S235 and K280), while SepM_control and SepM_D221N each harbored two such residues (LYS-280, and LYS-301 for SepM_control; ASP-278 and LYS-280 for SepM_G661A) close to the active center.

**Fig. 3:**
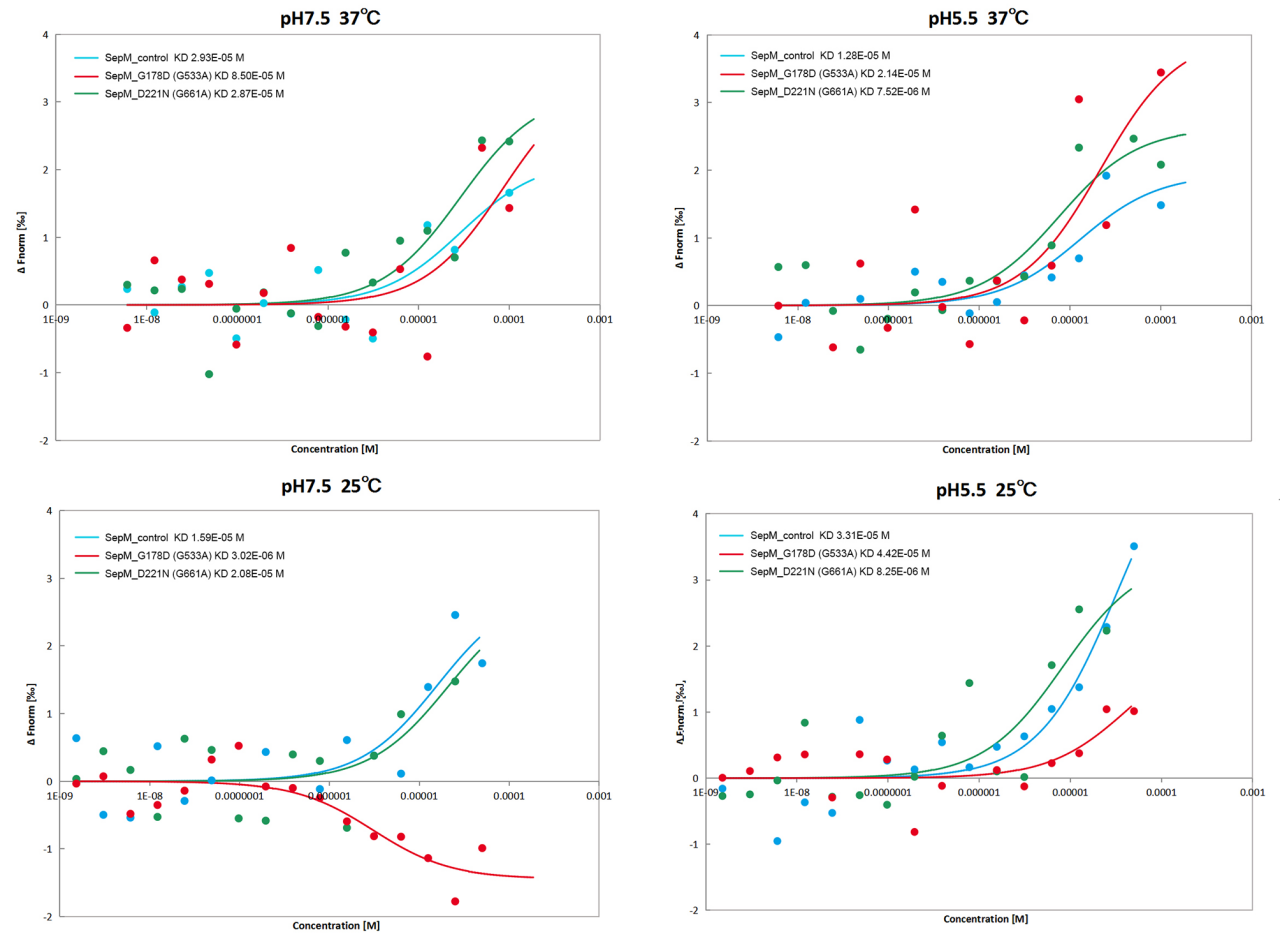
The affinity (KD) between SepMs (SepM_control, SepM_G178D, and SepM_D221N) and CSP-21

The expression and purification of SepMs (SepM_control, SepM_G178D, SepM_D221N) were illustrated in Fig. S2. Interactions between the three analyzed SepMs (SepM_control, SepM_G178D, SepM_D221N) and CSP-21 were assessed through an MST assay approach (Fig. 3). This analysis revealed that: (1) at 37 ℃ and pH 7.5, the respective affinities of these three proteins for CSP-21 were 29.3 ± 34.5 µM, 85.0 ± 240 µM, and 28.7 ± 24.1 µM; (2) at 37 ℃ and pH 5.5, the respective affinities of these three proteins for CSP-21 were 12.8 ± 10.3 µM, 21.4 ± 26.8 µM, and 7.52 ± 4.62 µM; (3) at 25 ℃ and pH 7.5, the respective affinities of these three proteins for CSP-21 were 15.9 ± 16.5 µM, 3.02 ± 2.27 µM, and 20.8 ± 47.1 µM; and (4) at 25 ℃ and pH 5.5, the respective affinities of these three proteins for CSP-21 were 33.1 ± 25.5 µM, 44.2 ± 86.8 µM, and 8.25 ± 8.65 µM.

## Discussion

Genetic variations in bacteria can alter their virulence phenotypes in a manner that may lower or raise the risk of disease. *S. mutans* is an important cariogenic bacterium. While *S. gordonii* is a dominant bacterium for early colonization of dental plaque, and is negatively correlated with the onset of dental caries. SepM plays an important role in the interaction between *S. mutans* and *S. gordonii*. In this study, we focused on SepM mutation in *S. mutans* clinical isolates and related function. Of these 286 *S. mutans* isolates, 149 and 137 isolates originated from the caries site and caries_free site, respectively. No correlation was found between the source of the isolates (caries or caries_free) and the inhibition of *S. mutans* against *S. gordonii* (*P* = 0.697). This may be because the inhibition of *S. gordonii* growth by *S. mutans* is not a direct factor in its cariogenic effect. The direct cariogenicity of *S. mutans* is to lower the local pH and induce demineralization [40]. Considering that the ability of *S. mutans* to inhibit *S. gordonii* is not a necessary factor for the occurrence of dental caries, we did not include the analysis of the source (caries or caries_free) of the strains in mutation analysis.

The G533A (G178D) mutation distribution differed most significantly when comparing the inhibitory and non-inhibitory groups, and the levels of SepM and associated regulatory protein and genes were thus analyzed in clinical isolates that did or did not harbor this G533A mutation. Gene expression level results suggested that none of the analyzed genes exhibited any differential expression patterns. As information is limited regarding appropriate internal reference proteins for use when studying *S. mutans*, protein levels were instead measured based on general protein quantification [34, 41]. Protein analyses revealed that the expression levels of the SepM and ComE proteins in the G533A group were elevated relative to the control group, although ComD protein levels did not vary between these groups, whereas the expression levels of the phosphorylated ComD in the G533A group were elevated relative to the control group. These indicate the possibility that the *sepM* G533A mutation may increase SepM protein expression without having any effect on *sepM* gene expression. The increased SepM will bind to more ComD proteins, inducing the latter to produce more phosphorylation levels, higher expression of ComE (phosphorylated ComE), and a stronger phenotype that inhibits the growth of *S. gordonii*. Unfortunately, we were unable to obtain clear phosphorylated ComE bands under various conditions. The expression levels of genes and their corresponding proteins in the same strains are not completely consistent. This does not rule out the possibility of another mechanism promote the translation of these genes or the discrepancy may be due to different protein stability.

Saswati Biswas et al. reported that when the mutation is located at the 18th and 19th amino acids of CSP-21, it can affect the activity of CSP-21 binding to SepM, while mutations at other amino acid sites do not significantly affect its binding to SepM [26]. The *comC* gene has a total length of 141 bp and encodes 46 amino acids. CSP-21 is located at the 76–138 bp of the *comC* gene in *S. mutans*. We also analyzed gene mutations in the CSP-21 fragment in these 286 *S. mutans* clinical isolates and found that the CSP-21 peptide segment only shared 6 mutations including 5 missense mutations and 1 synonymous mutation. However, there was no significant difference in the distribution of these missense mutations between the two groups. Considering that the site of these mutations is located at the first four amino acids of the CSP-21, which is far from the 18th and 19th amino acids of CSP-21, we think there is no difference in CSP-21 production by the various mutant strains in this study.

Molecular docking analyses were used to predict ligand interactions with the target binding site and to gauge the stability of this interaction [42]. However, protein flexibility was lacking in most instances [43], and the reliability of the resultant protein-peptide complexes is unclear. Molecular dynamics simulations can complement these docking analyses [44]. Here, both strategies were employed in parallel, revealing that the G533A mutation did not impact the stability of SepM binding to CSP-21, whereas it did impact the binding site of SepM where it interacts with this ligand. Specifically, the CSP-21 binding sites in the SepM_G533A protein included the LYS-50, ASP-278, LYS-301, and ILE-231 residues. SepM is a serine protease that localizes to the cell surface and harbors a Ser-Lys dyad (S235 and K280) active site [25]. The ASP-278 position is close to this active site K280 residue, and it may thus influence SepM activity.

In caries-free individuals, the oral pH is typically neutral [45, 46], while a pH of 5.5 is the critical threshold value for the demineralization of the enamel and caries development [47]. Accordingly, we assessed SepMs activity under these two pH levels. The equilibrium dissociation constant KD is used to describe the binding affinity between a given protein and its target ligand [48], with greater KD values indicating weaker affinity. Generally, the oral temperature is 37 ℃, and our results showed that there was no significant difference between the mutant protein and the control group under 37 ℃. But at 25 ℃ and a pH of 5.5, SepM_D221N exhibited high affinity for its target substrate, whereas the control SepM protein exhibited lower affinity. Similarly, at 25 ℃ and a pH of 7.5, the affinity of SepM_G178D for its cognate substrate was relatively high as compared to that of the control SepM. This suggests that *S. mutans* carrying mutant SepM proteins may play an important role in assisting directly cariogenic *S. mutans* strains in antagonizing other threatening species such as *S. gordonii* to gain competitive growth advantages on the tooth surface.

While this study offers new insight into the role that genetic modification in bacterial interactions, it is subject to limitations. In this study, we also tried to construct mutants in UA159, but the resultant strains were unstable and had poor vitality. We were puzzled whether the phenotypic changes were caused by mutations or because of the repeated freeze-thaw recovery and clonal screening selection. Relevant techniques need to be improved in the future to observe the specific effect of mutation on phenotype. Therefore, this study used protein prokaryotic expression and affinity experiments to compensate for the impact of gene mutations on the SepM protein. More experiments are needed to confirm the impact of mutations on the stability of the SepM protein.

## Conclusions

In summary, this study is the first to have systematically analyzed mutations present in *sepM* across *S. mutans* clinical isolates, enabling an assessment of the interplay between these *sepM* mutations and the inhibitory activity of *S. mutans* against *S. gordonii*. This research facilitated the verification of the effects of differentially distributed mutations on gene expression, protein expression, and SepM binding affinity for its substrates. Notably, these findings demonstrated that the *sepM* G533A (G178D) and G661A (D221N) mutations may impact protein translation levels or the stability of the protein, and the binding affinity of this protein for its substrate CSP-21, underscoring their relevance when exploring interactions between *S. mutans* and *S. gordonii* and the impact of these interactions on caries development from a genetic perspective.

### Electronic supplementary material

Below is the link to the electronic supplementary material.

Supplementary Material 1

Supplementary Material 2

Supplementary Material 3

Supplementary Material 4

Supplementary Material 5

## Data Availability

The datasets generated and/or analysed during the current study are available in the National Center for Biotechnology Information (NCBI) under the BioProject accession number PRJNA1016128.

## Abbreviations

S. mutans: Streptococcus mutans
S. gordonii: Streptococcus gordonii
PCR: Polymerase chain reaction
IPTG: Isopropyl-β-D-thiogalactopyranoside
MST: Microscale thermophoresis
IPD: Invasive pneumococcal disease

## References

[1] Force USPST, Davidson KW, Barry MJ, Mangione CM, Cabana M, Caughey AB, Davis EM, Donahue KE, Doubeni CA, Kubik M (2021). Screening and interventions to prevent Dental Caries in children younger than 5 years: US Preventive Services Task Force Recommendation Statement. JAMA.

[2] Innes NP, Clarkson JE, Douglas GVA, Ryan V, Wilson N, Homer T, Marshman Z, McColl E, Vale L, Robertson M (2020). Child Caries Management: a Randomized Controlled Trial in Dental Practice. J Dent Res.

[3] Amarasena N, Chrisopoulos S, Jamieson LM, Luzzi L. Oral health of Australian adults: distribution and Time Trends of Dental Caries, Periodontal Disease and tooth loss. Int J Environ Res Public Health 2021, 18(21). 10.3390/ijerph182111539 PMC8583389 34770052

[4] Nishijima T, Teruya K, Yanase M, Tamori Y, Mezaki K, Oka S (2012). Infectious endocarditis caused by Lactobacillus acidophilus in a patient with mistreated dental caries. Intern Med.

[5] Seow WK (2018). Early Childhood Caries. Pediatr Clin North Am.

[6] Tanzer JM, Thompson AM, Grant LP, Vickerman MM, Scannapieco FA (2008). Streptococcus gordonii’s sequenced strain CH1 glucosyltransferase determines persistent but not initial colonization of teeth of rats. Arch Oral Biol.

[7] Lemos JA, Palmer SR, Zeng L, Wen ZT, Kajfasz JK, Freires IA, Abranches J, Brady LJ. The Biology of Streptococcus mutans. Microbiol Spectr 2019, 7(1). 10.1128/microbiolspec.gpp3-0051-2018 PMC6615571 30657107

[8] Larsen MJ, Pearce EI (1997). A computer program for correlating dental plaque pH values, cH+, plaque titration, critical pH, resting pH and the solubility of enamel apatite. Arch Oral Biol.

[9] Palmer RJ, Gordon SM, Cisar JO, Kolenbrander PE (2003). Coaggregation-mediated interactions of Streptococci and actinomyces detected in initial human dental plaque. J Bacteriol.

[10] Nyvad B, Kilian M (1990). Comparison of the initial streptococcal microflora on dental enamel in caries-active and in caries-inactive individuals. Caries Res.

[11] Kreth J, Zhang Y, Herzberg MC (2008). Streptococcal antagonism in oral biofilms: Streptococcus sanguinis and Streptococcus gordonii interference with Streptococcus mutans. J Bacteriol.

[12] Chen L, Walker AR, Burne RA, Zeng L. Amino sugars reshape interactions between Streptococcus mutans and Streptococcus gordonii. Appl Environ Microbiol 2020, 87(1). 10.1128/AEM.01459-20 PMC7755246 33097515

[13] Mitrakul K, Vongsawan K, Sriutai A, Thosathan W (2016). Association between S. mutans and S. sanguinis in severe early childhood caries and caries-free children a quantitative real-time PCR analysis. J Clin Pediatr Dent.

[14] Bhaumik D, Salzman E, Davis E, Blostein F, Li G, Neiswanger K, Weyant RJ, Crout R, McNeil DW, Marazita ML et al. Plaque Microbiome in caries-active and caries-Free Teeth by Dentition. JDR Clin Trans Res 2022:23800844221121260. 10.1177/23800844221121260 PMC10725180 36154330

[15] Agnello M, Marques J, Cen L, Mittermuller B, Huang A, Chaichanasakul Tran N, Shi W, He X, Schroth RJ (2017). Microbiome Associated with severe caries in Canadian First Nations children. J Dent Res.

[16] AlEraky DM, Madi M, El Tantawi M, AlHumaid J, Fita S, AbdulAzeez S, Borgio JF, Al-Harbi FA, Alagl AS (2021). Predominance of non-streptococcus mutans bacteria in dental biofilm and its relation to caries progression. Saudi J Biol Sci.

[17] Qi F, Chen P, Caufield PW (2001). The group I strain of Streptococcus mutans, UA140, produces both the lantibiotic mutacin I and a nonlantibiotic bacteriocin, mutacin IV. Appl Environ Microbiol.

[18] Caufield PW, Childers NK, Allen DN, Hansen JB (1985). Distinct bacteriocin groups correlate with different groups of Streptococcus mutans plasmids. Infect Immun.

[19] Kreth J, Merritt J, Shi W, Qi F (2005). Co-ordinated bacteriocin production and competence development: a possible mechanism for taking up DNA from neighbouring species. Mol Microbiol.

[20] Hossain MS, Biswas I (2012). SMU.152 acts as an immunity protein for mutacin IV. J Bacteriol.

[21] Hossain MS, Biswas I (2011). Mutacins from Streptococcus mutans UA159 are active against multiple streptococcal species. Appl Environ Microbiol.

[22] van der Ploeg JR (2005). Regulation of bacteriocin production in Streptococcus mutans by the quorum-sensing system required for development of genetic competence. J Bacteriol.

[23] Tanzer JM, Thompson A, Sharma K, Vickerman MM, Haase EM, Scannapieco FA (2012). Streptococcus mutans out-competes Streptococcus gordonii in vivo. J Dent Res.

[24] Bikash CR, Hamry SR, Tal-Gan Y (2018). Structure-activity relationships of the competence stimulating peptide in Streptococcus mutans reveal motifs critical for membrane protease SepM recognition and ComD receptor activation. ACS Infect Dis.

[25] Hossain MS, Biswas I (2012). An extracelluar protease, SepM, generates functional competence-stimulating peptide in Streptococcus mutans UA159. J Bacteriol.

[26] Biswas S, Cao L, Kim A, Biswas I (2016). SepM, a streptococcal protease involved in Quorum sensing, displays strict substrate specificity. J Bacteriol.

[27] Zimmermann P (2006). The prevalence and significance of PDZ domain-phosphoinositide interactions. Biochim Biophys Acta.

[28] Jemth P, Gianni S (2007). PDZ domains: folding and binding. Biochemistry.

[29] Liu S, Li H, Zhang K, Guo Z, Zheng Q, Hu F, Zhang W, Sun Y, Guan JC. Phenotypic and genetic characteristics of Streptococcus mutans isolates from site-specific dental plaque in China. J Med Microbiol 2021, 70(3). 10.1099/jmm.0.001313 33459586

[30] Liu S, Sun Y, Liu Y, Hu F, Xu L, Zheng Q, Wang Q, Zeng G, Zhang K (2022). Genomic and phenotypic characterization of Streptococcus mutans isolates suggests key gene clusters in regulating its interaction with Streptococcus gordonii. Front Microbiol.

[31] Shklair IL, Keene HJ (1974). A biochemical scheme for the separation of the five varieties of Streptococcus mutans. Arch Oral Biol.

[32] Nomura R, Nakano K, Taniguchi N, Lapirattanakul J, Nemoto H, Gronroos L, Alaluusua S, Ooshima T (2009). Molecular and clinical analyses of the gene encoding the collagen-binding adhesin of Streptococcus mutans. J Med Microbiol.

[33] Liu S, Li X, Guo Z, Liu H, Sun Y, Liu Y, Wang Q, Liao S, Zhang K. A Core Genome Multilocus Sequence Typing Scheme for Streptococcus mutans. *mSphere* 2020, 5(4). 10.1128/mSphere.00348-20 PMC7343978 32641425

[34] Lei L, Zhang B, Mao M, Chen H, Wu S, Deng Y, Yang Y, Zhou H, Hu T (2020). Carbohydrate metabolism regulated by antisense vicR RNA in Cariogenicity. J Dent Res.

[35] Yang J, Yan R, Roy A, Xu D, Poisson J, Zhang Y (2015). The I-TASSER suite: protein structure and function prediction. Nat Methods.

[36] Zheng W, Zhang C, Li Y, Pearce R, Bell EW, Zhang Y. Folding non-homologous proteins by coupling deep-learning contact maps with I-TASSER assembly simulations. Cell Rep Methods 2021, 1(3). 10.1016/j.crmeth.2021.100014 PMC8336924 34355210

[37] Jabir NR, Rehman MT, Alsolami K, Shakil S, Zughaibi TA, Alserihi RF, Khan MS, AlAjmi MF, Tabrez S (2021). Concatenation of molecular docking and molecular simulation of BACE-1, gamma-secretase targeted ligands: in pursuit of Alzheimer’s treatment. Ann Med.

[38] Yan Y, Zhang D, Zhou P, Li B, Huang SY (2017). HDOCK: a web server for protein-protein and protein-DNA/RNA docking based on a hybrid strategy. Nucleic Acids Res.

[39] Cuellar J, Astrand M, Elovaara H, Pietikainen A, Siren S, Liljeblad A, Guedez G, Salminen TA, Hytonen J. Structural and biomolecular analyses of Borrelia burgdorferi BmpD reveal a substrate-binding protein of an ABC-Type Nucleoside Transporter Family. Infect Immun 2020, 88(4). 10.1128/IAI.00962-19 PMC7093131 31988175

[40] Lin Y, Chen J, Zhou X, Li Y (2021). Inhibition of Streptococcus mutans biofilm formation by strategies targeting the metabolism of exopolysaccharides. Crit Rev Microbiol.

[41] Lei L, Stipp RN, Chen T, Wu SZ, Hu T, Duncan MJ (2018). Activity of Streptococcus mutans VicR is modulated by antisense RNA. J Dent Res.

[42] Agrawal P, Singh H, Srivastava HK, Singh S, Kishore G, Raghava GPS (2019). Benchmarking of different molecular docking methods for protein-peptide docking. BMC Bioinformatics.

[43] Alonso H, Bliznyuk AA, Gready JE (2006). Combining docking and molecular dynamic simulations in drug design. Med Res Rev.

[44] Santos LHS, Ferreira RS, Caffarena ER (2019). Integrating Molecular Docking and Molecular Dynamics simulations. Methods Mol Biol.

[45] Peker S, Kargul B, Tanboga I, Tunali-Akbay T, Yarat A, Karakoc F, Ersu R, Dagli E (2015). Oral health and related factors in a group of children with cystic fibrosis in Istanbul, Turkey. Niger J Clin Pract.

[46] Veeresh T, Mujahid A, Deepu P, Sivaprakash R (2019). Correlation between Dermatoglyphics, Dental Caries and Salivary pH: an Invivo Study. Ethiop J Health Sci.

[47] Struzycka I (2014). The oral microbiome in dental caries. Pol J Microbiol.

[48] Yamada M, Miyawaki A, Saito K, Nakajima T, Yamamoto-Hino M, Ryo Y, Furuichi T, Mikoshiba K. The calmodulin-binding domain in the mouse type 1 inositol 1,4,5-trisphosphate receptor. Biochem J 1995, 308 (Pt 1)(Pt 1):83–88. 10.1042/bj3080083 PMC1136846 7755592

