# Supplementary figures and images for: SepM mutation in Streptococcus mutans clinical isolates and related function analysis
Source: BMC Oral Health. 2024 Jun 25;24:730. doi: 10.1186/s12903-024-04436-x (PMC11197336; doi:10.1186/s12903-024-04436-x)

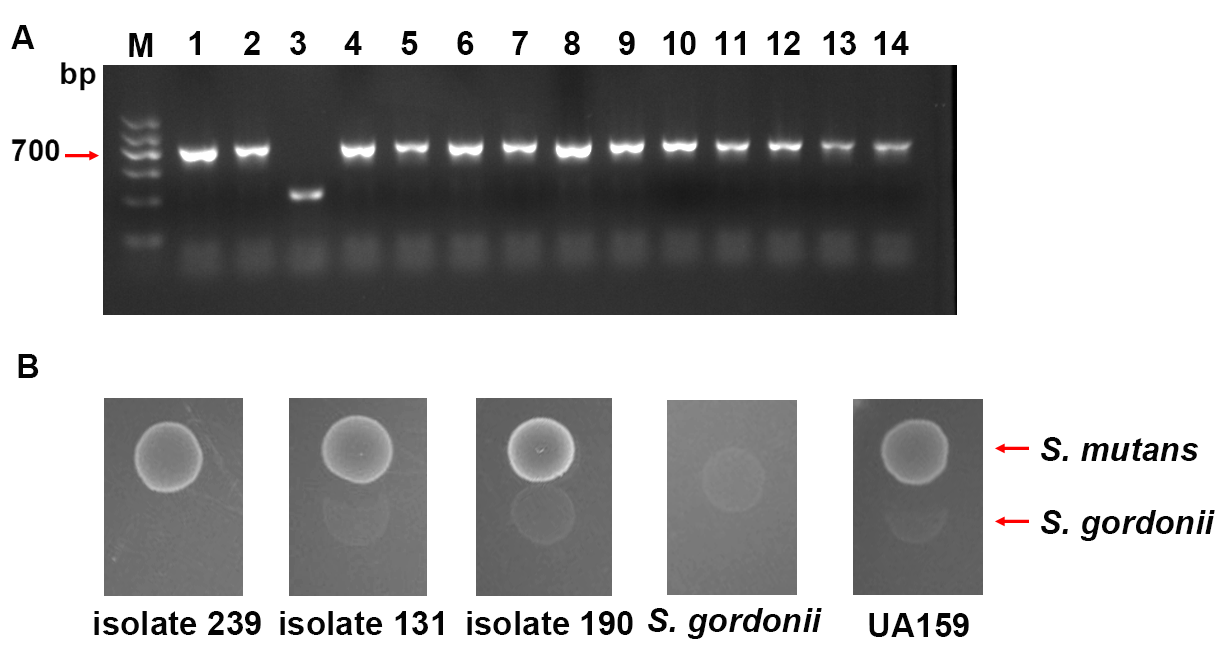

Supplement: Supplementary file 1 — Supplementary Material 1 [file 12903_2024_4436_MOESM1_ESM.tif]

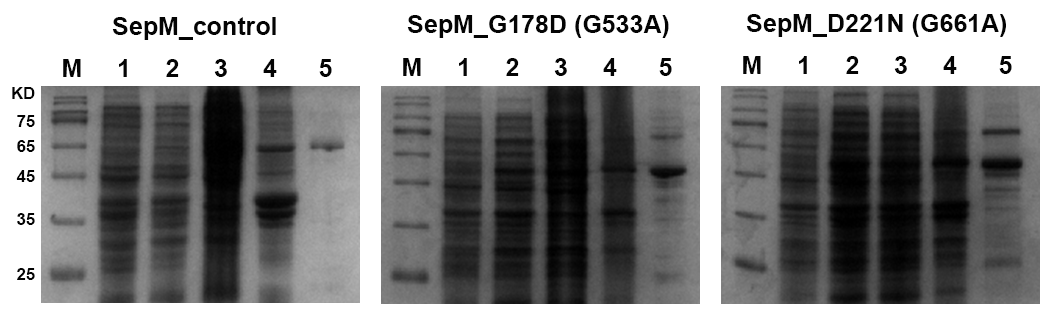

Supplement: Supplementary file 2 — Supplementary Material 2 [file 12903_2024_4436_MOESM2_ESM.tif]

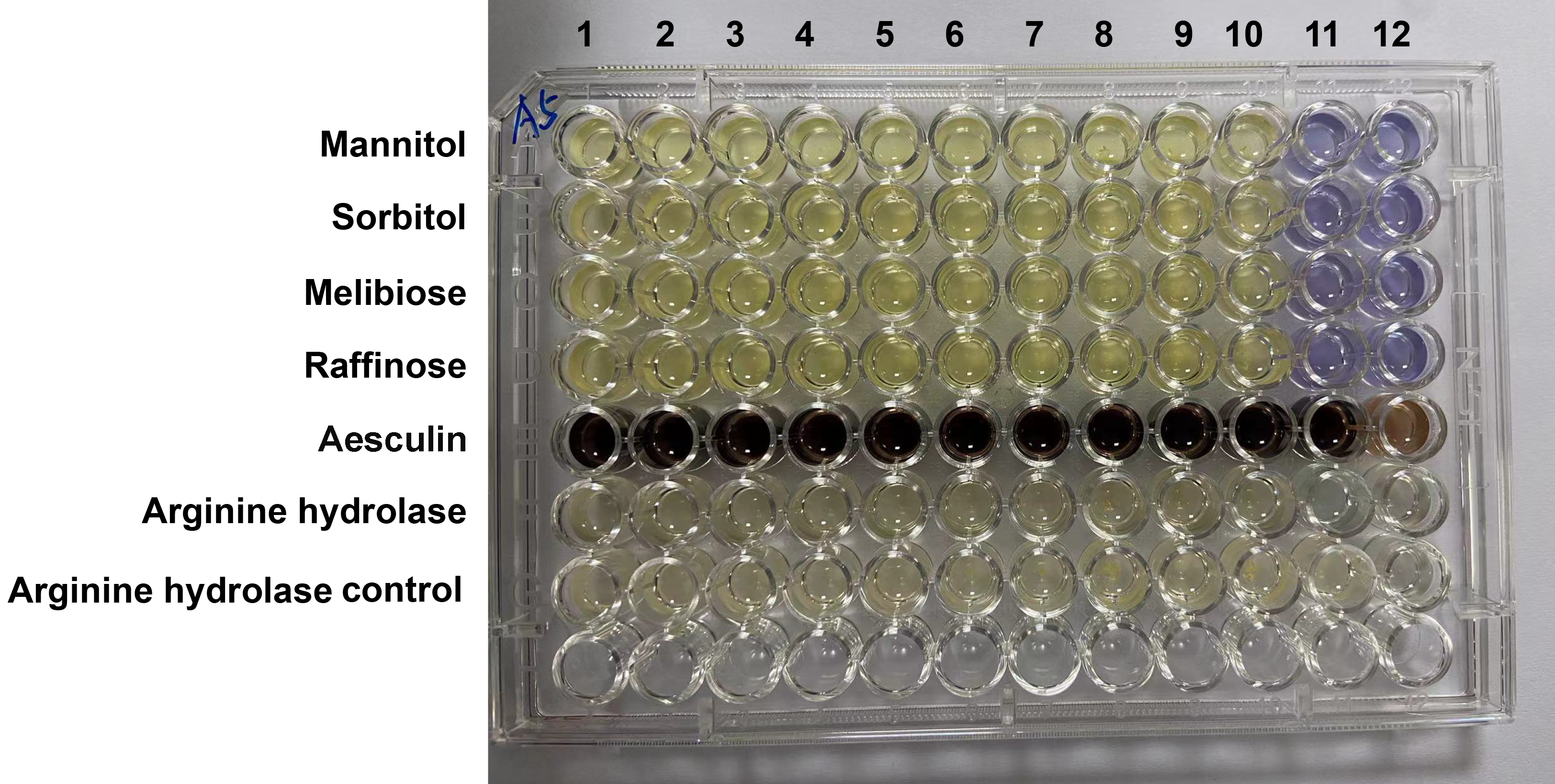

Supplement: Supplementary file 4 — Supplementary Material 4 [file 12903_2024_4436_MOESM4_ESM.tif]
