# Supplementary material for: SepM mutation in Streptococcus mutans clinical isolates and related function analysis
Source: BMC Oral Health. 2024 Jun 25;24:730. doi: 10.1186/s12903-024-04436-x (PMC11197336; doi:10.1186/s12903-024-04436-x)
Supplement: Supplementary file 3 — Supplementary Material 3 [file 12903_2024_4436_MOESM3_ESM.docx]

**Appendix table 1** The relationship between CSP-21 mutations and the virulence of *S. mutans* against *S. gordonii*

| Gene mutation | Reference codon | Mutation codon | Amino mutation | Mutation type | Distribution of mutation (n, %) | | |
| --- | --- | --- | --- | --- | --- | --- | --- |
|  |  |  |  |  | Inhibitory group  (n=114) | Non-inhibitory group  (n=172) | *P* value |
| G1A | AGC | AAC | S1N | Missense | 0 (0.0) | 3 (1.74) | 0.278 |
| G1C | AGC | ACC | S1T | Missense | 3 (2.63) | 1 (0.58) | 0.305 |
| G5A | GGA | GAA | G2E | Missense | 1 (0.88) | 0 (0.0) | 0.399 |
| G8C | AGC | ACC | S3T | Missense | 8 (7.02) | 4 (2.33) | 0.070 |
| T11A | CTA | CAA | L4Q | Missense | 0 (0.0) | 2 (1.16) | 0.519 |
| A12G | CTA | CTG | L4L | Synonymous | 11 (9.65) | 2 (1.16) | 0.001 |
