# Supplementary material for: SepM mutation in Streptococcus mutans clinical isolates and related function analysis
Source: BMC Oral Health. 2024 Jun 25;24:730. doi: 10.1186/s12903-024-04436-x (PMC11197336; doi:10.1186/s12903-024-04436-x)

Figure legends for Figure S1-S3

Figure S1 (A) The C serotype validation of the *S. mutans* clinical isolates. The marker corresponds to 1200, 900, 700, 500, 300 and 100 bp from top to bottom. Lane 1 was *S. mutans* UA159 (positive control) and lanes 2-28 were clinical isolates. Lanes 2, 4-14 were isolates with C serotype (727 bp); (**B**) The activity of *S. mutans* clinical isolates against *S. gordonii* ATCC 10558. The clearing zones in *S gordonii* domain represent *S. mutans*'s activity against *S. gordonii*. Isolates 239, 131, and 190 represent clinical strains that completely inhibit, partially inhibit, and completely fail to inhibit the growth of *S. gordonii*. *S. mutans* UA159 was used as a positive reference.

Figure S2 Expression and purification of SepM_control, SepM_G178D, and SepM_D221N. Lane 1, cells expressing SepM without any induction; Lane 2, cells expressing SepM after induction; Lane 3, SepM expression in supernatants after cell lysis; Lane 4, SepM expression in precipitates after cell disruption; Lane 5, purified recombinant SepM.

Figure S3 The reaction of clinical strains with mannitol, sorbitol, raffinose, melibiose, aesculin, arginine hydrolase and arginine hydrolase control. Line 1 represents positive control (S. mutans UA159), lines 2-11 represent clinical strains, line 12 represents negative control (0.9% saline).

Gels with membrane edges visible in Figure 1 B

SepM G533A G533A_free G533A G533A_free

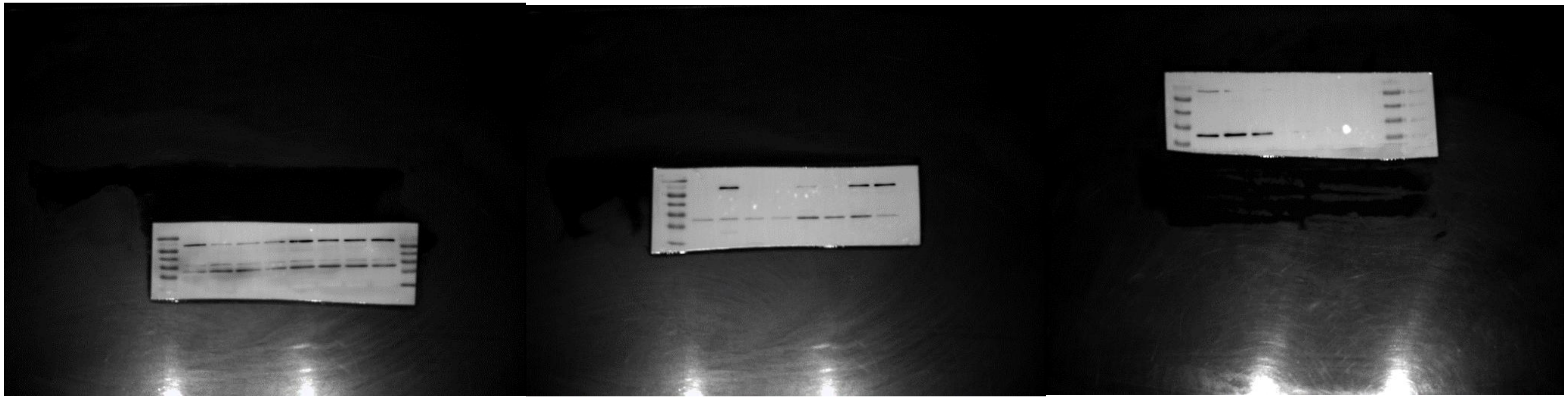

ComD G533A G533A_free G533A G533A_free

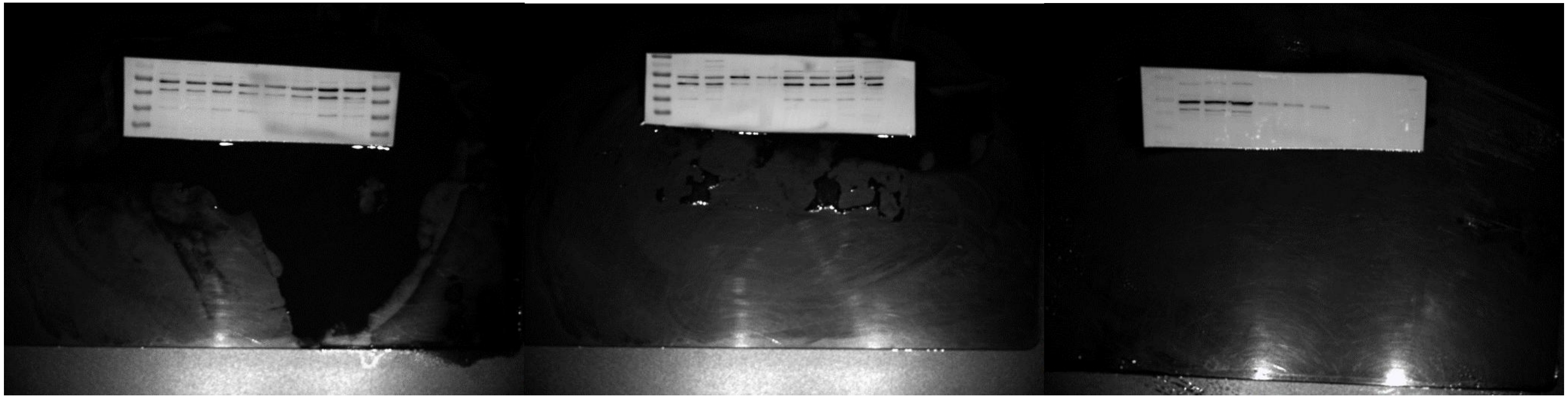

ComD-P G533A G533A_free G533A G533A_free

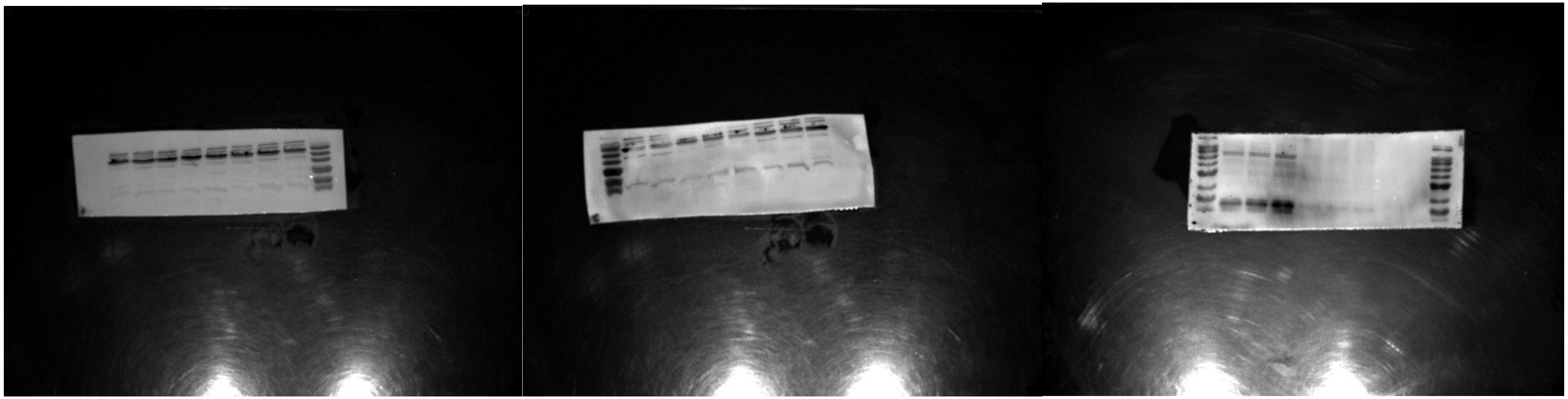

ComE G533A G533A_free G533A G533A_free

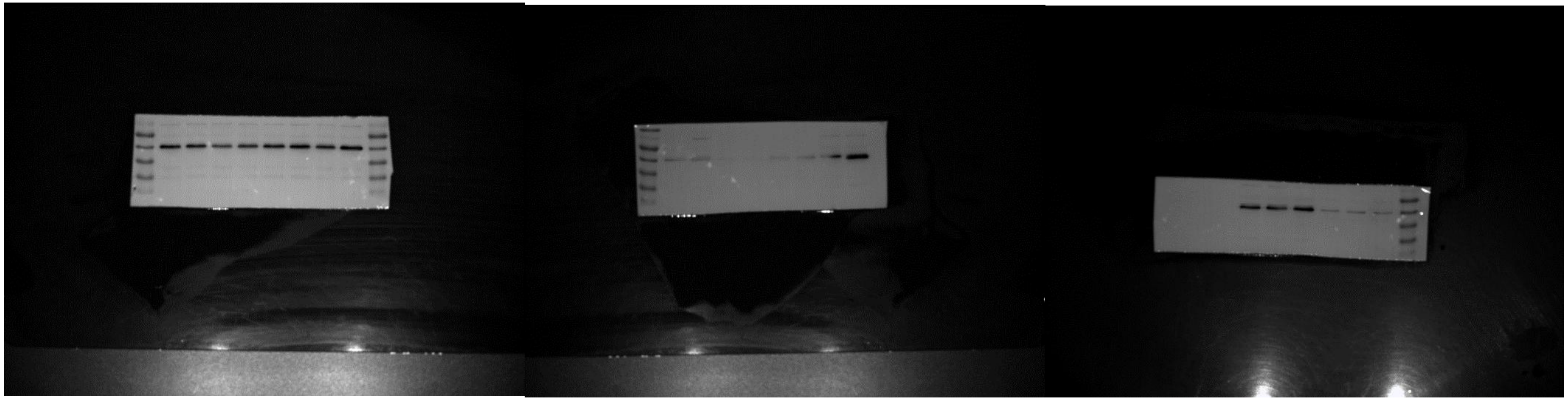

The upper half of the agarose gel plot is the original image of Fig S1 A

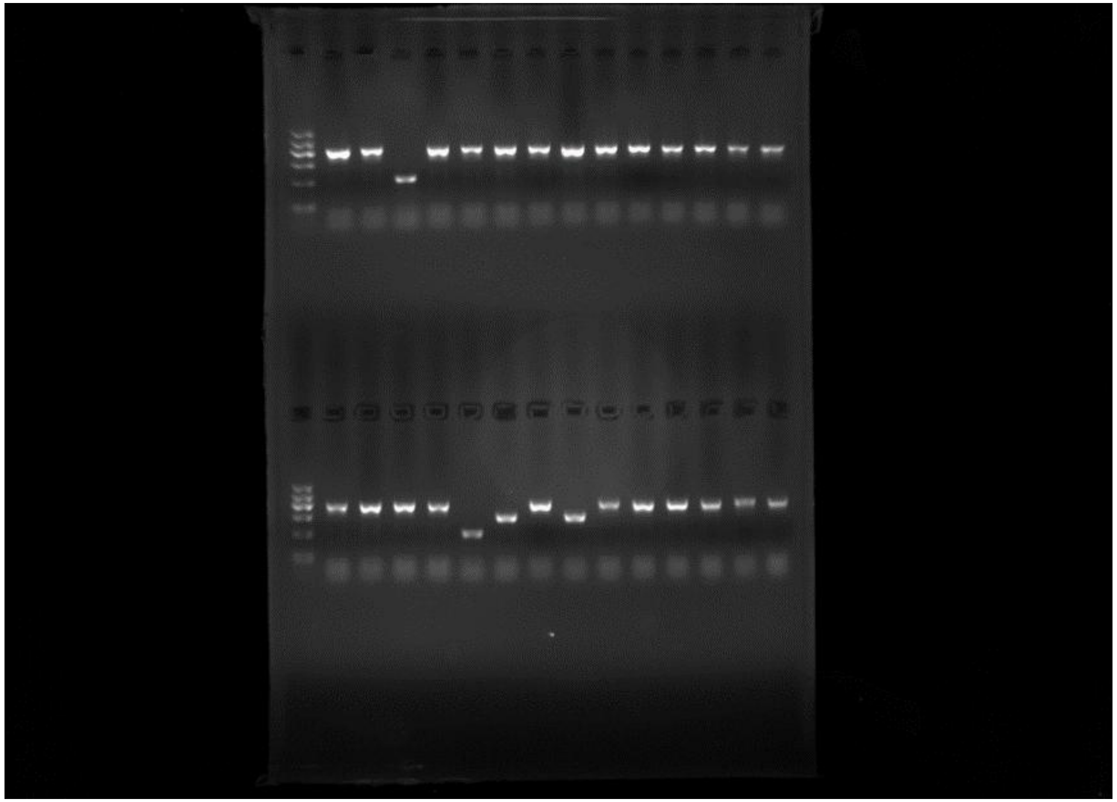

Gels with membrane edges visible in Figure S2

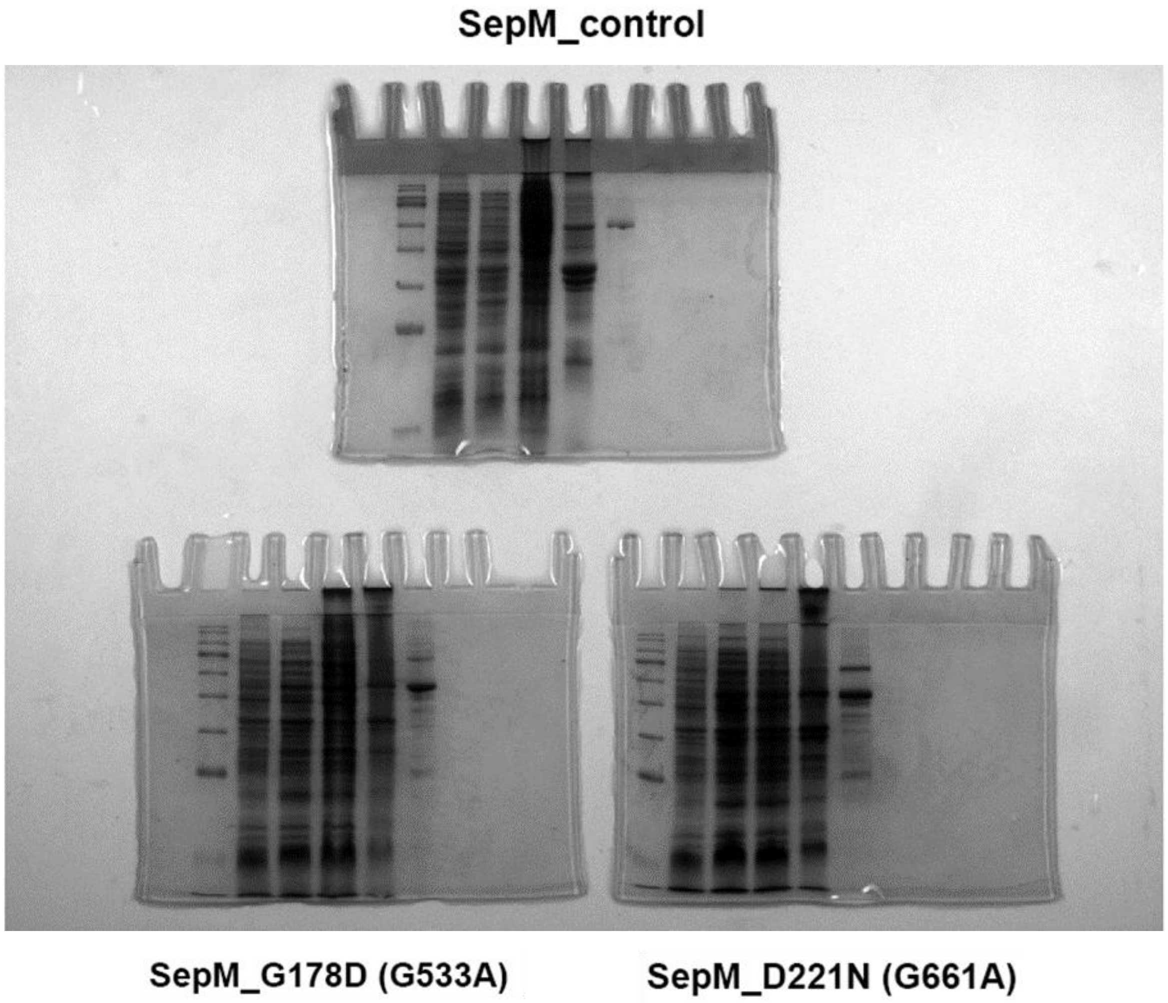

Supplement: Supplementary file 5 — Supplementary Material 5 [file 12903_2024_4436_MOESM5_ESM.pdf]
